# A fatal case of neonatal onset multiple acyl-CoA dehydrogenase deficiency caused by novel mutation of ETFDH gene: case report

**DOI:** 10.1186/s13052-022-01356-w

**Published:** 2022-09-05

**Authors:** Loredana De Pasquale, Petronilla Meo, Francesco Fulia, Antonio Anania, Valerio Meli, Antonina Mondello, Maria Tindara Raimondo, Viviana Tulino, Maria Sole Coletta, Caterina Cacace

**Affiliations:** Azienda Sanitaria Provinciale di Messina - Neonatal Intensive Care Unit, Barone Romeo Hospital, Patti, ME Italy

**Keywords:** ETF, ETFDH, Glutaric aciduria type II, Multiple acyl-CoA dehydrogenase deficiency, Case report

## Abstract

**Background:**

Multiple acyl-CoA dehydrogenase deficiency (MADD) or glutaric aciduria type II is an extremely rare autosomal recessive inborn error of fatty acid beta oxidation and branched-chain amino acids, secondary to mutations in the genes encoding the electron transfer flavoproteins A and B (ETFs; ETFA or ETFB) or ETF dehydrogenase (ETFDH). The clinical manifestation of MADD are heterogeneous, from severe neonatal forms to mild late-onset forms.

**Case presentation:**

We report the case of a preterm newborn who died a few days after birth for a severe picture of untreatable metabolic acidosis. The diagnosis of neonatal onset MADD was suggested on the basis of clinical features displaying congenital abnormalities and confirmed by the results of expanded newborn screening, which arrived the day the newborn died. Molecular genetic test revealed a homozygous indel variant c.606 + 1 _606 + 2insT in the ETFDH gene, localized in a canonical splite site. This variant, segregated from the two heterozygous parents, is not present in the general population frequency database and has never been reported in the literature.

**Discussion and conclusion:**

Recently introduced Expanded Newborn Screening is very important for a timely diagnosis of Inherited Metabolic Disorders like MADD. In some cases which are the most severe, diagnosis may arrive after symptoms are already present or may be the neonate already died. This stress the importance of collecting all possible samples to give parents a proper diagnosis and a genetic counselling for future pregnacies.

## Background

Multiple acyl-CoA dehydrogenase deficiency (MADD) or glutaric aciduria type II (GA II; MIM #231680) is an extremely rare (ie, < 1:250.000) autosomal recessive inborn error of fatty acid beta oxidation and branched-chain amino acid, lysine, and tryptophan metabolism [1]. The disorder originates from mutations in the genes encoding the electron transfer flavoproteins A and B (ETFs; ETFA or ETFB) or ETF dehydrogenase (ETFDH), also known as ETF-ubiquinone oxidoreductase. ETF serves as a specific electron acceptor that transfers electrons to ubiquinone in the respiratory chain in a process catalysed by ETFDH. At least 9 distinct dehydrogenases depend on the ETF pathway. The metabolic defect results in impaired ATP biosynthesis, hypoketosis, excessive lipid accumulation in tissues, and reduced gluconeogenic capacity [2]. The severity of the disease depends on the location and nature of mutations. Truncation or null mutations in any of these genes can result in severe phenotypes, while missense ones tend to occur in the milder forms of the disorder [3]. On the basis of the age of onset and of the clinical presentation, three main subtypes have been distinguished: I) neonatal onset with congenital anomalies; II) neonatal onset without anomalies; III) late-onset with relatively mild phenotype [4]. The first two forms share life-threatening course with high mortality rate during the first few days of life, mainly due to untreatable metabolic acidosis, non-ketotic hypoglicemia and hyperammonemia. The clinical course in late-onset form is usually milder with high clinical heterogeneity that explains its difficult identification especially when the symptoms arise in adult life [5]. The diagnosis is usually supposed soon after the birth via population newborn bloodspot screening (NBS) but requires confirmation by genetic investigations [6, 7]; however, despite early detection, the prognosis is poor in type I-II. On this topic, we report a fatal case of type I GA II caused by a novel mutation of the ETFDH gene.

## Case presentation

A male preterm infant was born from healthy non-consanguineous parents at 32 + 6 weeks of gestation, via caesarean section delivery for foetal bradycardia and podalic presentation. The pregnancy was characterized by oligohydramnios and presence of voluminous cerebral cyst both detected at 28 + 2 weeks of gestation. Neonatal weight was 1230 g (<10th centile), length 40.0 cm (10th centile), occipital-frontal circumference 27.5 cm (<3th centile). Apgar score was 7/8 at 1 and 5 minutes, respectively. The patient was immediately transferred to the neonatal intensive care unit of our hospital because of moderate respiratory distress (FiO_2_ > 0.3) requiring respiratory support by continuous positive airway pressure (CPAP) ventilation. Hemogasanalysis revealed a condition of metabolic acidosis (PH 7.11, PCO2 42 mmHg, PO2 60 mmHg, BE-16.2 mmol/L, HCO3- 13.3 mmHg, lactates 6,2 mmol/L). Laboratory investigations revealed hyperchloremia (> 110 mmol/L) and normal values of glycemia and ammoniemia. Clinical features were dominated by facial dysmorphisms (ogival palate, low-set ears), bilateral syndattilia of the 2th and 4th toes and empty scrotum. Minimal enteral feeding with formula and total parenteral nutrition were started. On day of life (DOL) 2 the persistence of hyperchloremic metabolic acidosis refractory to correction with bicarbonate was complicated by high values of liver transaminases, hyperglycemia (250 mg/dl) with glycosuria. Urine was negative for ketones. The infant underwent transfusion with packed red cells because of anaemia of the prematurity (hemoglobin 8,6 g/dl). B-mode abdominal ultrasound showed normal sized but echogenic kidneys with loss of cortico-medullary differentiation. On DOL 3, onset of leukopenia with neutropenia (< 0,5 × 10^9^/L), requiring infusion of granulocyte stimulating factor, was observed. The metabolic acidosis was unresponsive to sodium bicarbonate up to 5 mEq/kg every 6 hours. A “sweaty feet” smell of the urine was noted. Urine samples were collected to search for organic acids, and blood samples were used to perform the expanded newborn bloodspot screening and the determination of plasma acylcarnitine profiles to identify an eventual congenital metabolic disease. Peripheral blood samples from proband and parents were also collected for genetic testing. On DOL 4 the general conditions dramatically worsened, with severe respiratory failure and rapid deterioration of renal function, with contraction of the diuresis and increase of blood urea (171 mg /dl), serum creatinine (1.8 mg/dl) and ammonemia (225 mmol/L). Despite tracheal intubation, invasive mechanical ventilation and supportive care, the newborn died on DOL 5. Autopsy was not performed because of parents’ refusal. The results of NBS, along with plasma and urine exams, arrived on DOL 5. The results from the plasma acylglycines analysis were abnormal (Table 1), showing elevated concentrations of long and medium chain acylcarnitines species, including C4, C5, C5DC/C6OH, C6, C14, C14:2, C14OH, C16, C16:1, C161OH, C18, together with increased values of arginine, citrulline, glycine, methionine, proline, tyrosine (Table 2) supporting the diagnosis of GA II. Urine organic acids analysis revealed abnormal urinary excretion of lactic, glutaric, adipic, isovaleric and parahydroxyphenyllatic acids together with raised levels of isovalerylglycina. The results were strongly suggestive for the diagnosis of MADD and the results of NBS confirmed it. Genetic analysis carried out using next-generation sequencing revealed a homozygous mutation in the ETFDH gene of the infant (c606 + 1_606 + 2insT) in a canonical site of splicing. This previously unreported mutation was inherited from the parents, each of whom carried only one heterozygous variant. The same mutation was detected in mother’s sister. The diagnosis of autosomal recessive GA II was confirmed, and genetic counseling was therefore offered to parents to inform them about the percentage of risk to have an affected child in case of a new pregnancy.

**Table 1:** Acylcarnitine profile of the patient. Numbers out of reference range are in bold

Analyte Name	Measured value	Target value	Reference range	Units
C0- Free carnitine	4.913	26.025	7.65-44.4	umol/L
C2- Acetylcarnitine	3.729	26.64	8.81-44.47	umol/L
C3-Propionylcarnitine	0.184	2.46	0-4.92	umol/L
C3DC-Malonylcarnitine	0.081	0.185	0-0.37	umol/L
C4-n-butyryl−/isobutyrylcarnitine	**2.743**	0.315	0-0.63	umol/L
C5-Isovaleryl−/2-Methylbutyrylcarnitine	**3.802**	0.12	0-0.24	umol/L
C5:1-Tiglylcarnitine	0.01	0.01	0–0.02	umol/L
C5DC-Glutaryl/3-Hydroxydecanoylcarnitine	**0.559**	0.075	0-0.15	umol/L
C6-Hexanoylcarnitine	**0.124**	0.05	0-0.1	umol/L
C8-Octanoylcarnitine	0.091	0.065	0-0.13	umol/L
C10-Decanoylcarnitine	0.14	0.1	0-0.2	umol/L
C10:1-Decenoylcarnitine	0.045	0.035	0-0.07	umol/L
C10:2-Decadienoylcarnitine	0.003	0.005	0-0.01	umol/L
C12-Dodecanoylcarnitine	0.226	0.14	0-0.28	umol/L
C12:1-Dodecenoylcarnitine	0.076	0.11	0-0.22	umol/L
C14-Tetradecanoylcarnitine	**1.011**	0.215	0-0.43	umol/L
C14:1-Tetradecenoylcarnitine	0.295	0.155	0-0.31	umol/L
C14:2-Tetradecadienoylcarnitine	**0.082**	0.02	0-0.04	umol/L
C16-Hexadecanoylcarnitine	**9.634**	4.41	1.59-7.23	umol/L
C16:1-Hexadecenoylcarnitine	**1.178**	0.21	0-0.042	umol/L
C18-Octadecanoylcarnitine	**3.173**	1.135	0.44-1.83	umol/L
C18:1-Octadecenoylcarnitine	**3.295**	1.265	0-2.53	umol/L
C18:2-Linoleylcarnitine	**0.868**	0.17	0-0.34	umol/L

**Table 2:** Amino acids profile of the patient. Numbers out of reference range are in bold

Analyte Name	Measured value	Target value	Reference range	Units
Alanine	371.874	258.68	0-517.36	umol/L
Arginine	**59.701**	14.595	2.03-27.16	umol/L
Citrulline	**43.409**	15.085	6.36-23.81	umol/L
Glutamine	**712.633**	554.695	0-1109.39	umol/L
Glutamate	123.221	240.22	0-480.44	umol/L
Glycine	400.043	94.81	0-786.9	umol/L
Leucine/isoleucine/Proline	284.251	94.81	0-189.62	umol/L
Methionine	**36.801**	15.38	6.46-24.3	umol/L
Ornithine	**297.692**	101.73	0-203.46	umol/L
Phenylalanine	55.204	37.3	0-74.6	umol/L
Proline	**904.92**	138.295	68.16-208.43	umol/L
Tyrosine	**175.164**	82.595	0-165.19	umol/L
Valine	**233.089**	103.475	0-206.95	umol/L

## Discussion and conclusions

Acyl-coenzyme A (CoA) dehydrogenases (ACDHs) are a family of mitochondrial flavoproteins actively involved in mitochondrial fatty acid β-oxidation (FAO), branched chain amino acid (BCAA) catabolism and amino acid catabolism [8]. These enzymes require oxidised flavin adenine dinucleotide (FAD) as cofactor. FAD continuous re-oxidation is provided by the concerted action of ETFs and ETFDH [9]. Mutations in the genes codifying for these flavoproteins give rise to a variety of different inborn errors of metabolism, so-called MADD, characterized by the impairment of the FAO and BCAA catabolic pathways responsible for downstream effects that explain the typical phenotype and the peculiar laboratory findings observed in our patient [2, 5, 6, 9, 10]. FAO is deeply involved in ATP production pathway in the heart, kidney and skeletal muscle, together with glycolysis. In MADD, its defective activity causes a compensatory hyper activation of glycolysis and glycogenolysis to ensure the right ATP provision necessary for the correct functioning of cellular processes [11]. The dysfuction of Glutaryl CoA Dehydrogenases due to mutation in ETFs or ETFDH leads to increased accumulation of glutaric acids, which is responsible for metabolic acidosis that doesn’t benefit from aggressive treatment with bicarbonate boluses [12]. The defective mitochondrial fatty acid beta-oxidation processes negatively influence acetyl-COA production, with accumulation of large amounts of fatty acyl-COA species and of the corresponding acylglycine and -carnitine coniugates, increased lipid accumulation in the liver and muscles, high blood methionine and phenylalanine levels, and decreased ketone body and blood glucose levels [11]. Lipid accumulation is observed in the tissues including the liver, heart and renal tubular epithelium, which use fatty acids as a primary source of energy. Colevas et al. speculated that the malformations might be the consequence of an accumulation of toxic metabolites that is not corrected by placental transfer [13]. Formal clinical diagnostic criteria for MADD have not been established. The diagnosis is suspected on the basis of the combination of clinical and supportive specific and non-specific laboratory findings and requires confirmation by identification of biallelic pathogenic variants in ETFA, ETFB, or ETFDH genes. Our patient had growth retardation, with reduced OFC, ogival palate, low-set ears, bilateral syndattilia of the 2th and 4th toes and empty scrotum as well as loss of cortico-medullary differentiation of the kidney. This is coherent with the description of congenital alterations occurring in type I MADD including dysmorphic facial features (high anterior hairline, wide nasal bridge, short nose with anteverted nares and long philtrum, tented upper lip, midface retrusion), single palmar creases, rocker-bottom feet, hypospadias with or without chordee in males. Renal malformation with large cystic kidneys and antenatal oligohydramnios, cardiomyopathy and hepatomegaly may also be seen. Non-specific laboratory findings include nonketotic or hypoketotic hypoglycemia, with blood glucose often less than 45 mg/dL, hyperammonemia, elevated liver transaminases (AST, ALT). The absence of ketones at urinalysis in the setting of hypoglicemia is an important laboratory marker that may contribute to clinically differentiate MADD-related hyperlactatemia from other conditions linked to primitive metabolic defects of the Krebs’ cycle before NBS results have become available. The condition of hyperglycemia recorded in our patient instead of hypoglycemia may be the consequence of the parenteral nutrition in the first days of life. Strongly suggestive for MADD is the typical plasma acylcarnitine profile evidenced on blood dried spot by the tandem mass spectrometry analysis, with elevations of C4, C5, C5DC, C6, C8, C10, C12, C14:1, C16, and C18:1 acylcarnitine [5, 6, 10, 14] The urine organic acid analysis reveals a combination of increased excretion of glutaric-, ethylmalonic-, adipic-, suberic-, sebacic-, dodecanedioic-, 2-hydroxyglutaric-, 3 -, and 5- hydroxyhexanoic acid, in conjunction with C4 and C5 glycine conjugates i.e. isobutyrylglycin, isovalerylglycine, hexanoylglycine, suberylglycine. The urinary excretion of hydroxy isovaleric acid explains the unpleasant feet smell of the patient’s urine. However, the diagnosis may be challenging in late-onset cases since the biochemical abnormalities may be mild, atypical or only detectable during metabolic decompensations [15].MADD differential diagnoses include disorders of riboflavin metabolism with similar biochemical findings or overlapping phenotypic features, like the severe congenital anomalies associated neonatal-onset form of carnitine palmitoyl transferase II deficiency, and the neonatal transient MADD-like illness. The latter lacks birth defects and is caused by a maternal riboflavin deficiency sustained by a heterozygous pathogenic variant with secondary neonatal riboflavin deficiency, that dramatically improve after riboflavin supplementation [16]. Molecular genetic testing is mandatory to confirm the diagnosis. MADD is inherited in an autosomal recessive manner, caused by loss-of-function variants. A recent systematic review has identified 36, 19 and 280 pathogenic variants of ETFA, ETFB and ETFDH genes, respectively [11]. Homozygous or compound heterozygous null pathogenic variants or pathogenic variants that severely affect mRNA expression or stability result in a complete loss of protein expression or function and cause the most severe form of MAD. Instead, pathogenic variants involving the active site and/or pathogenic splice site variants give rise to very low residual enzyme activity, leading to neonatal onset without congenital malformations. Missense mutations not affecting the active site, mRNA expression, or stability have relatively high residual activity, and subsequently cause milder, late-onset disease (type III) [3]. Next-generation sequencing genetic analysis revealed a homozygous indel variant c.606 + 1 _606 + 2insT in the ETFDH gene, localized in a canonical splite site. This indistinct variant, segregated from the two heterozygous parents, is not present in the general population frequency database and has never been reported in the literature. In silico analyses predict that this variant is probably damaging to protein structure and it can be assigned to class IV according to standard guidelines of the American College of Medical Genetics and Genomics [17]. In this case, the diagnosis of MADD by expanded neonatal screening arrived the day the newborn died, simultaneously to the results of typical plasma acylcarnitine profile and urine organic acid analysis, which were consistent with the diagnosis. This case underlines the importance of expanded newborn screening in the diagnosis of congenital metabolic diseases which may present complex clinical pictures at birth. For that reason, the collecting of blood sample in the first days of life is essential and should be always performed. Moreover, the recognition of new mutations in the ETFDH gene, such as in this case, may improve also the quality of genetic counseling for parents who desire a new pregnancy.

## Data Availability

The datasets used and analyzed during the current study are available from the corresponding author on reasonable request.

## Abbreviations

MADD: Multiple acyl-CoA dehydrogenase deficiency
GA II: Glutaric aciduria type II
EFT: Electron transfer flavoproteins
ETFH: Electron transfer flavoproteins dehydrogenase
CPAP: Continuous positive airway pressure
DOL: Day of life
ACDH: Acyl-coenzyme A (CoA) dehydrogenase
FAO: Fatty acid β-oxidation
BCAA: Branched chain amino acid
